# What can speech production errors tell us about cross-linguistic processing in bilingual aphasia? Evidence from four English/Afrikaans bilingual individuals with aphasia

**DOI:** 10.4102/sajcd.v62i1.111

**Published:** 2015-06-26

**Authors:** Diane Kendall, Lisa Edmonds, Anine van Zyl, Inge Odendaal, Molly Stein, Anita van der Merwe

**Affiliations:** 1 VA RR & D Puget Sound DVA Medical Center, Speech and Hearing Sciences, University of Washington, United States of America; 2 Fulbright, University of Pretoria, Pretoria, South Africa; 3 Department of Biobehavioral Sciences, Speech Language Pathology, Teachers College, Columbia University, United States of America; 4 Speech-Language Pathology and Audiology, University of Pretoria, South Africa

## Abstract

**Introduction:**

The aim of this study is contribute to clinical practice of bilinguals around the globe, as well as to add to our understanding of bilingual aphasia processing, by analysing confrontation naming data from four Afrikaans/English bilingual individuals with acquired aphasia due to a left hemisphere stroke.

**Methods:**

This is a case series analysis of four Afrikaans/English bilingual aphasic individuals following a left cerebrovascular accident. Error analysis of confrontation naming data in both languages was performed. Research questions were directed toward the between language differences in lexical retrieval abilities, types of errors produced and degree of cognate overlap.

**Results:**

Three of the four participants showed significantly higher naming accuracy in first acquired language (L1) relative to the second acquired language (L2) and the largest proportion of error type for those three participants in both L1 and L2 was omission. One of the four participants (linguistically balanced) showed no between language accuracy difference. Regarding cognate overlap, there was a trend for higher accuracy for higher cognate words (compared to low).

**Discussion:**

This study showed that naming performance in these four individuals was reflective of their relative language proficiency and use patterns prior to their stroke. These findings are consistent with the hierarchical model, in normal bilingual speakers and with persons with bilingual aphasia.

## Introduction

Aphasia is a chronic, pervasive and often debilitating impairment of language that is most often a consequence of stroke in the left cerebral hemisphere. Speech-language pathologists strive to provide effective clinical rehabilitative services to remediate language impairments in persons with aphasia (PWA); with increasing prevalence of bilingualism in the world, special consideration to bilingual aphasia is imperative. Bilingual aphasia is a complex topic largely because of the heterogeneity within and across bilingual speakers due to varying manners of acquisition, patterns of use and relative abilities across languages (Grosjean, 1998). Thus, speech-language clinicians need to not only be cognisant of the potential variability of language backgrounds of people with aphasia but also knowledgeable regarding the mechanisms of lexical representation and processing in bilingual aphasia so that effective diagnostic and rehabilitative strategies can be employed.

Understanding the psycholinguistic mechanisms involved in bilingual word production can be viewed similarly to the activation and selection mechanisms in monolingual word production. Dell’s (1986) interactive activation theory of word processing (Dell, Schwartz, Martin, Saffran & Gagnon, 1997) states that word retrieval processes are a result of three layers of activity: semantic, lemma and phoneme. Connections between the layers allow bidirectional spread of activation (e.g. semantics ↔ lemma ↔ phonemes). For example, during confrontation naming of the word cat, visual analysis allows for identification of the picture with activation to corresponding semantic units or features, with bidirectional spread of information to lemma and phonological layers. This cascading process also occurs in reverse during comprehension with bottom-up activated phonological nodes spreading connections to lemma nodes, then to corresponding semantic nodes.

Similar to Dell’s interactive model, models of bilingual language representation and processing also account for a conceptual or semantic layer (shared between languages for most concepts), a lexical layer (with distinct lexical representations for each of the languages) (Kroll & Stewart, 1994) and a phoneme layer (with a certain degree of overlap between the phonological systems of the languages depending upon how closely the languages are sublexically related) (Costa, 2005). Essentially, in bilingual word processing, activation of the semantic system that is shared between languages spreads to both lexicons. This flow of activation is target language nonspecific regardless of the language in which the task is being performed (Costa, 2005; Edmonds & Kiran, 2006). For example, in a bilingual Afrikaans and English speaker, activation of the concept of ‘the piece of furniture in the dining room used to eat upon’ results in simultaneous activation of ‘tafel’ and ‘table’. Regardless of target-language output, this conceptual or semantic activation spreads to two distinct lexicons, then on to phonologic encoding of each of the language-specific words.

**FIGURE 1:**
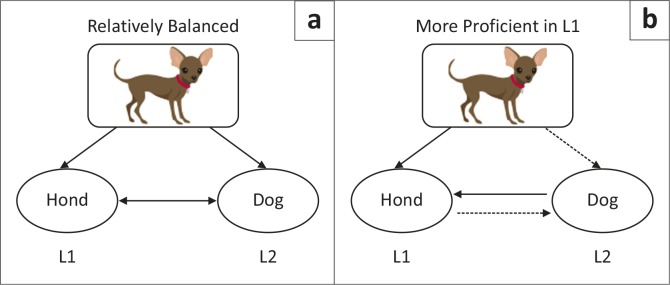
Model of bilingual language.

Factors known to influence the extent of interconnectivity and production abilities of the two languages in bilingual speakers include the linguistic task itself (e.g. narrative vs naming) and typological characteristics of each language (e.g. tone vs nontonal). Further, characteristics inherent to bilingual speakers that can influence production are related to patterns and relative use across languages (e.g. Kroll & Stewart, 1994). According to the hierarchical model, which we present as our working model for bilingualism, the strength of connections between the semantic system and each lexicon is dependent on use and proficiency, which are largely related to each other. With respect to lexical retrieval, a person’s more proficient language corresponds to that which is used more. As a result, the connections between the semantic system and the lexicon of the more proficient language are stronger, facilitating faster retrieval and a larger lexicon. Further, connections between the two lexicons (i.e. translation of words, e.g. ‘hond’ to ‘dog’) are also dependent on relative proficiency across languages, as translation from the less proficient language to the more proficient language is faster and more accurate than the reverse; these patterns are thought to correspond to relative strength of connections (mixed model, De Groot, 1992) (see Figure 1).

Cognate status (words with the same meaning that have some degree of overlapping phonology or orthography) is a structural element of language that has also been shown to impact word processing in bilinguals (e.g. Costa, Caramazza & Sebastian-Galles, 2000; Costa, Santesteban & Caño, 2005; Gollan & Acenas, 2004). That is, the extent to which phonological and orthographic similarities exist between two languages can influence the speed to which the words are processed and produced. For example, hart (Afrikaans) and heart (English) sharing the same number of syllables and initial consonants (high cognate overlap), whilst stoel (Afrikaans) and chair (English) share only the same number of syllables without vowel or consonant overlap (low cognate overlap). Costa *et al.* (2000) investigated cognate status on the speed of processing and found that during a picture naming task, naming latencies were faster for pictures of cognates than for pictures with noncognate names. The results were interpreted in the context of spread of activation such that words with overlapping cognates share semantic and phonological activation from two sources (target and nontarget language representations), whilst words without overlapping cognates only receive activation from a single language, rendering slower and less efficient production.

In a later article, Costa *et al.* (2005) posited and provided further evidence from the literature for the assumption that cognate translations in the nontarget language receive activation from the semantic system, along with the corresponding phonology, and that phonological segments send back activation to the lexical segments in both languages that they are connected to. However, different language combinations have differing degrees of cognates depending on phonological overlap, contact and borrowed words.

Although there is evidence of general facilitation effect of cognates, bilinguals’ relative proficiency across languages impacts that effect. In typical bilingual adults, it appears that the benefits of cognates is observed in the second acquired language (L2) of persons with relatively balanced abilities across languages (e.g. Gollan, Fennema-Notestine, Montoya & Jernigan, 2007; Roberts & Deslauriers, 1999), whereas there is no effect of cognate status in persons with a more proficient language. In other words, relatively balanced bilinguals exhibit similar accuracy across first acquired language (L1) and L2 in general, although they are more accurate with cognates in L2. In bilinguals with a more proficient language, their accuracy is lower in L2 in general, with no cognate effect.

Less is known about cognate production in persons with bilingual aphasia. Roberts and Deslauriers (1999) report that persons with bilingual aphasia reported higher accuracy for naming pictures with cognate words than non-cognate words in the language learned second in a group of French/English participants who reported high use and proficiency in both languages, which were learned before age 10. Other studies directly examining cognate effects in multilingual aphasia are more difficult to interpret because they were single case studies, involved polyglots (rather than bilinguals) and provided limited information on the cognates used in their studies (Ferrand & Humphreys, 1996; Stadie, Springer, De Bleser & Burk, 1995). Because confrontation naming is an integral component of aphasia testing, and often an outcome measure for treatment, more information is needed regarding the relationship between cognate status of items and proficiency and use patterns in persons with bilingual aphasia.

Most individuals living in South Africa speak more than one language (Statistics South Africa, 2012). Dutch and English were the first official languages of South Africa from 1909–1925. Between 1984 and 1994 the two official languages of South Africa were English and Afrikaans (Thompson, 2001). Since 1994, South Africa has had 11 official languages, nine of which are African Bantu languages, whilst two, English and Afrikaans, are Germanic languages. Because English and Afrikaans are West Germanic languages, their linguistic characteristics overlap considerably. Both languages are considered more analytic than synthetic; that is, they make use of relatively few bound morphemes (Barber, 1999). English and Afrikaans are also inflectional languages, implying that their bound morphemes are not invariable and each morpheme may indicate more than one characteristic, in contrast to agglutinative languages (such as Finnish and isiZulu) or isolating languages (such as Chinese and Vietnamese), whose bound morphemes are invariable and only indicate one characteristic (Barber, 1999). Although both English and Afrikaans make use of intonation to distinguish sentence or utterance types, neither uses syllabic tone to indicate differences in word meaning (that is, tone is not phonemic in either English or Afrikaans) (Kuiper & Scott Allan, 1996; Odendaal, 1989).

The high incidence of bilingual English/Afrikaans speakers in South Africa as well as the amount of overlap of these two Germanic languages presents a unique challenge to clinicians working with individuals with aphasia in that region. Furthermore, in addition to informing clinical practice of bilinguals around the globe, as well as adding to our understanding of bilingual aphasia processing, we have analysed confrontation naming data from four Afrikaans/English bilingual individuals with acquired aphasia due to a left hemisphere stroke. In a retrospective case series analysis, we examined the differential performance in lexical retrieval abilities between L1 and L2, types of errors produced and, finally, if performance was influenced by degree of cognate overlap. We also considered relative premorbid language use as reported by participants to contextualise our findings. The following research questions were posed:

1. Is there a significant difference in confrontation naming abilities between L1 and L2 as measured by accuracy?
2. Is there a difference between L1 and L2 in the types of errors produced as measured by raw number and proportion of errors?
3. Is there a difference in spoken word accuracy between L1 and L2 for high and low overlapping cognates?

## Methods

### Participants

#### Ethical considerations

Ethical clearance was obtained from the local university in South Africa. Individuals were recruited by solicitation from clinical speech-language pathologists (SLPs) who work in Pretoria hospitals, clinics and rehabilitation centres. Data were collected by two speech pathology students (third and fourth authors).

Inclusion criteria included the presence of a left hemisphere stroke, the presence of aphasia determined by a clinical SLP, bilingual speaker of English/Afrikaans with one language indicated by the participant as the predominant language and adequate hearing thresholds to conduct all tasks. The participants had to be capable of using full sentences or phrases in L1 during communication to ensure adequate verbal ability to perform the task. Exclusion criteria included severe aphasia, severely compromised receptive language ability, psychiatric illness, degenerative neurological disease, chronic medical illness, severe impairment in vision or hearing and knowledge or use of a third language.

The four participants were on average 73 years of age (SD 11.7) and ranged between four years and two months post stroke onset. There were two men and two women, all were right-handed and were bilingual in Afrikaans and English (see Table 1).

**TABLE 1:** Participant demographics

Participant	Age	Occupation	Gender	Time post stroke onset	Age of exposure for first (L1) and second language (L2) learned	Relative proficiency or use information by participant report
1	72	University lecturer (PhD)	Male	3 months	L1 Afrikaans (birth) L2 English (3 years)	Fully bilingual since early age with equal mastery of both languages.
2	84	Homemaker	Female	2 months	L1 English (birth) L2 Afrikaans (4 years)	High frequency English use. Exposed to Afrikaans through community and school.
3	57	Engineer (Four-year degree)	Male	3 months	L1 Afrikaans (birth) L2 English (8 years)	MoreAfrikaans use and proficiency. Exposed to English through TV and school. Usedboth languages at work.
4	76	Homemaker (High school degree)	Female	3 years and 5 months	L1 Afrikaans (birth) L2 English (‘as a young child of approximately 8 years’)	High frequency Afrikaans use, though she reported good English vocabulary.

Participant 1 (P1), male, age 72 was three months post stroke onset, had a university education and worked premorbidly as a university lecturer. He stated that he was fully bilingual since an early age, with acquisition of Afrikaans first (L1) and English later (L2) at the age of three. He was exposed to English through family and school and predominately spoke English in his occupational setting.

Participant 2 (P2), female, age 84 was two months post stroke onset, completed Grade 12 and worked as a homemaker. She stated that she learned English first in the home (L1) and was exposed to Afrikaans from the age of four years (L2). She indicated that she was exposed to both English and Afrikaans in both the community and school and reported to have mastered both languages equally. For approximately 20 years pre-onset she was mainly exposed to English (L1) and used only English at home.

Participant 3 (P3), male, age 57 was three months post stroke onset, had a four-year university degree and worked as an engineer. He stated that his first language was Afrikaans (L1) and that his second language, English (L2), was acquired at approximately eight years, predominately through formal classes in school and by watching the television. He regarded himself as quite proficient in both languages, using English and Afrikaans interchangeably at work.

Participant 4 (P4), female, age 76 was three years and five months post stroke onset, had a Grade 12 education and worked as a homemaker. She stated that her first and most used language was Afrikaans (L1) and that she was exposed to English (L2) at age eight from formal lessons at school. She stated that she had a good English vocabulary prior to her stroke; however, she did not use English as much as Afrikaans in her daily life.

### Stimuli

In order to elicit confrontation naming, colour pictures corresponding to 40 one-syllable, two-syllable, and three-syllable nouns were selected in English and Afrikaans. High and low frequency words were selected from http://www.wordcount.org (see Table 2). Word pairs (in these two languages) had the same number of syllables. To determine name agreement, each picture was presented on a 14 cm by 22 cm card to five healthy Afrikaans/English bilingual adults who named the pictures with 100% accuracy and 100% agreement.

**TABLE 2:** Stimuli used in experiment with degree of cognate overlap.

Afrikaans	English	Initial Sound	# Syllables	Consonants	Vowels
		3 Same consonant		3. >70% overlap	
		2 Same vowel	2 Same #	2. 50-70% overlap	2. > 80% vowel overlap
		1 Similar sound (*e.g. same sound class or one of consonant cluster*)	1 Diff by only 1 syllable	1. < 50% overlap	1. 50-80% overlap
		0 Complete mismatch	0 Diff by more than 1 syllable	0. No overlap	0. no vowel overlap
man	man	3	2	3	0
hart	heart	3	2	3	1
kinders	children	0	2	1	1
tafel	table	3	2	1	1
tamatie	tomato	3	2	2	1
slak	snail	3	2	1	0
baba	baby	3	2	3	0
boek	book	3	2	3	0
telefoon	telephone	3	2	3	1
wol	wool	3	2	3	0
skoenlapper	butterfly	0	2	0	0
blaar	leaf	1	2	1	0
aarbeie	strawberries	0	1	1	1
hoed	hat	3	2	2	0
atleet	athlete	3	2	1	1
hond	dog	0	2	1	0
roomys	ice cream	0	2	0	0
perd	horse	0	2	0	0
oog	eye	0	2	0	0
robot	robot	3	2	3	2
deur	door	3	1	3	0
kat	cat	3	2	3	0
venster	window	0	2	1	0
reenboog	rainbow	3	2	2	0
rekenaar	computer	0	2	1	0
sop	soup	3	2	3	0
skeermes	razor	0	2	1	0
kam	comb	3	2	3	0
pynappel	pineapple	3	2	3	1
zip	zip	3	2	3	2
helikopter	helicopter	3	2	3	1
ster	star	3	2	3	0
mikrogolf	microwave	3	2	1	1
neus	nose	3	2	2	0
oorbel	earring	0	2	0	0
huis	house	3	2	3	0
pizza	pizza	3	2	3	2
knoop	knot	3	2	2	0
stoel	chair	0	1	0	0
legkaart	puzzle	0	2	0	0

### Overlapping cognate analysis

A 10-point scale to quantify the degree of phonological overlap between translation word pairs was employed (see Kohnert, Windsor & Miller, 2004 for details regarding development of scale). This cognate scale indexes four different features: initial sound, number of syllables, percentage of overlapping consonants and percentage of overlapping vowels. A specific rating scale is employed to further describe each of the four features. Initial sound is scored between 0 and 3 points (3 = same consonant, 2 = same vowel, 1 = similar sound, 0 = complete mismatch). Number of syllables was scored between 0 and 2 points (2 = same number of syllables, 1 = different by only one syllable, 0 = different by more than one syllable). Consonants were scored between 0 and 3 points (3 = >70% consonant overlap, 2 = 50%–70% overlap, 1 = <50% overlap, 0 = no overlap). Finally, vowels were scored between 0 and 2 points (2 = >80% overlap, 1 = 50%–80% overlap, 0 = no overlap). All scores for each word were tallied. For the purposes of this study, we deemed that a word was judged to have high overlap if it was between 8 and 10, medium overlap between 5 and 7, low overlap between 1 and 4 and no overlap if 0. See Table 2 for results of cognate scores.

### Data collection procedures

Participants were seated in a quiet room and stimulus cards were randomised and presented one at a time without any cues. The participants were requested to first name all objects in the set of 40 pictures in L1. After completion of the set of pictures in L1 they were again presented with the pictures, which were again randomised, and asked to name the pictures in L2. L1 was consistently probed first to control the possible influence of uncertainty or higher demands in L2. Instructions were provided in both languages (English for the English naming and Afrikaans for the Afrikaans naming). Participant responses were not constrained with a time limit. Participant responses were audio recorded and later scored for overall accuracy, total number of phonemes correctly produced and error type by three members from the motor speech laboratory at the University of Pretoria (first, fifth and sixth authors).

### Outcome measure analysis

The first research question addressed confrontation picture naming whole word accuracy. The second question addressed total number of phonemes correct and types of errors made. The final question looked at naming accuracy as a function of high, medium and low overlapping cognate status. Methods and procedures for each of these outcome measures are described below.

### Error coding procedures

Digital recordings of participant responses were scored, by consensus, by three trained SLPs (first and last two authors), two of whom are proficient Afrikaans/English bilingual speakers. The English speaking only SLP scored just the English responses. Only the participants’ first responses were coded. Correct response included the correct lexical item only. Fillers such as ‘um’ and ‘I don't know’ were not counted. Incorrect responses were coded for error type using five categories:

1. phonologic (P) – substitutions, additions, transpositions, omissions.
2. semantic (S) – related within language (SR-w: ‘child’ for ‘girl’), unrelated within language (SU–w: ‘grape’ for ‘house’), related across language (SR-a: English ‘colt’ for Afrikaans ‘perd’, which means horse), unrelated across language (SU-a: English ‘horse’ for Afrikaans ‘stoel’, which means chair).
3. mixed (M) – real words with a phonologic relationship to the target.
4. omission (O) – includes circumlocutions and non-responses.
5. neologism (N) – nonwords that were not phonologically related to the target.

Direct translations were coded (T), as well as translations with phonologic errors (Tp).

### Data analysis

To answer research question 1, paired-samples unequal variance t tests comparing confrontation naming accuracy between L1 and L2 were performed. For the error analysis questions, paired-samples t tests compared L1 and L2 for raw number of each error type and error type proportions (proportion of each error type relative to total numbers of errors made). For the cognate question, percentage accuracy was documented for high, medium and low overlap cognate words and paired-samples t tests were used to compare L1 and L2 differences.

## Results

### Reliability of outcome measure scoring

Consensus reliability on 25% of the corpus resulted in 98% point-to-point reliability on accuracy scores and 83% reliability on error type coding.

### Overall accuracy

**FIGURE 2:**
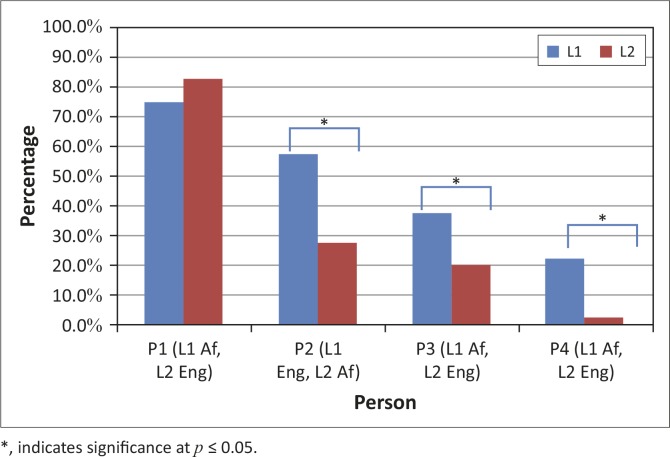
Confrontation naming accuracy for four bilingual – Afrikaans (Af) and English (Eng) – persons with aphasia (Research question #1).

The first research question asked if there was a significant difference in confrontation naming abilities between L1 and L2 as measured by accuracy. Results for P1 showed 75% (L1) and 83% (L2) accuracy, resulting in no difference (*p* = 0.209). Results for P2 were 58% (L1) and 28% (L2), showing a significant between language difference (*p* = 0.038). Results for P3 were 38% (L1) and 20% (L2) accurate, showing statistical significance (*p* = 0.047). Finally, results for P4 were 23% (L1) and 2.5% (L2) accurate, which was significantly different (*p* = 0.047) (see Figure 2).

### Error type

**FIGURE 3:**
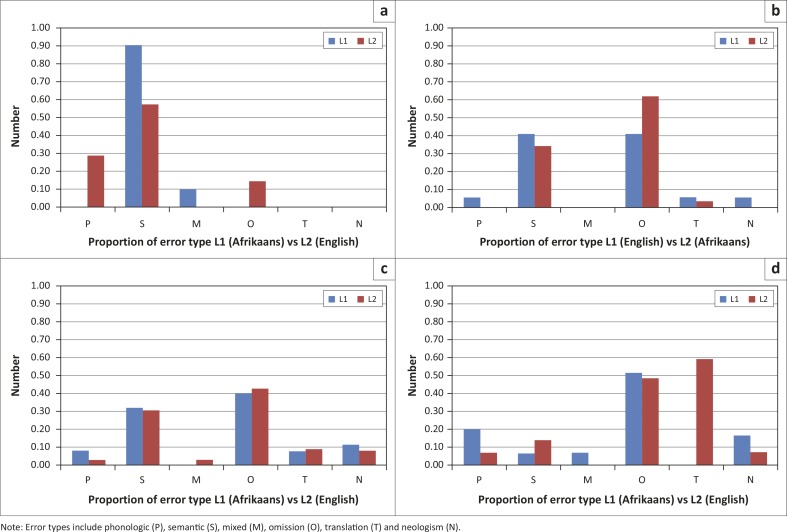
P1, P2, P3 and P4: Proportion of error types for L1 versus L2 (Research question #2)

The second research question asked if there was a significant difference for each participant between L1 and L2 in the number and types of errors produced as measured by raw number and proportion of errors. Results for each participant, for L1 and L2, are described and are shown in Figure 3.

P1 (L1) had 10 raw errors (out of 40 words). Nine errors were semantic and one mixed. The L1 semantic errors were further subdivided into eight within language-related errors and one within language-unrelated error. Proportion of errors were 0.90 semantic and 0.10 mixed. In L2, he had seven total errors (out of 40 words). Four errors were semantic, two phonologic and one omission. The L2 semantic errors were further subdivided into three within language-related errors and one within language-unrelated error. Proportion of errors was 0.29 phonologic, 0.57 semantic and 0.14 omission.

P2 (L1) had 17 raw errors (out of 40 words). Seven errors were semantic, 1 phonologic, 7 omission, 1 translation and 1 neologism. The L1 semantic errors were further subdivided into 5 within language-related, 1 within language-unrelated and 1 across language-related error categories. Proportion of errors was 0.41 semantic, 0.06 mixed, 0.06 translation and 0.06 neologism. In L2, she had 29 errors (out of 40 words). Ten errors were semantic, 18 omission and 1 translation. The L2 semantic errors were further subdivided into 7 within language related, 2 within language unrelated and 1 across language related categories. Proportion of errors was 0.34 semantic, 0.62 omission and 0.03 translation.

P3 (L1) had 25 raw errors (out of 40 words). Eight errors were semantic, two phonologic, 10 omission, two translation and three neologism. The L1 semantic errors were further subdivided into two across language-unrelated, three within language-unrelated, one across language-related and two within language-related categories. Proportion of errors were 0.08 phonologic, 0.32 semantic, 0.40 omission, 0.08 translation and 0.12 neologism. In L2, he had 32 errors (out of 40 words). Ten errors were semantic, one phonologic, one mixed, 14 omission, three translation and three neologism. The L2 semantic errors were further subdivided into two across language-unrelated, four within language-related, one across language-related and one within language-related categories. Proportion of errors was 0.31 semantic, 0.03 phonologic, 0.03 mixed, 0.43 omission, 0.09 translation and 0.09 neologism.

P4 (L1) had 31 raw errors (out of 40 words). Two errors were semantic, six phonologic, two mixed, 16 omission and five neologism. The L1 semantic errors were further subdivided into one within language-unrelated and one within language-related categories. Proportion of errors was 0.19 phonologic, 0.06 semantic, 0.07 mixed, 0.48 omission and 0.16 neologism. In L2, she had 39 errors (out of 40 words). Four errors were semantic, two phonologic, 14 omission, 17 translation and two neologism. The L2 semantic errors were further subdivided into one within language-unrelated and three across language-related categories. Proportion of errors was 0.14 semantic, 0.07 phonologic, 0.48 omission, 0.59 translation and 0.07 neologism.

### Cognate effect

**FIGURE 4:**
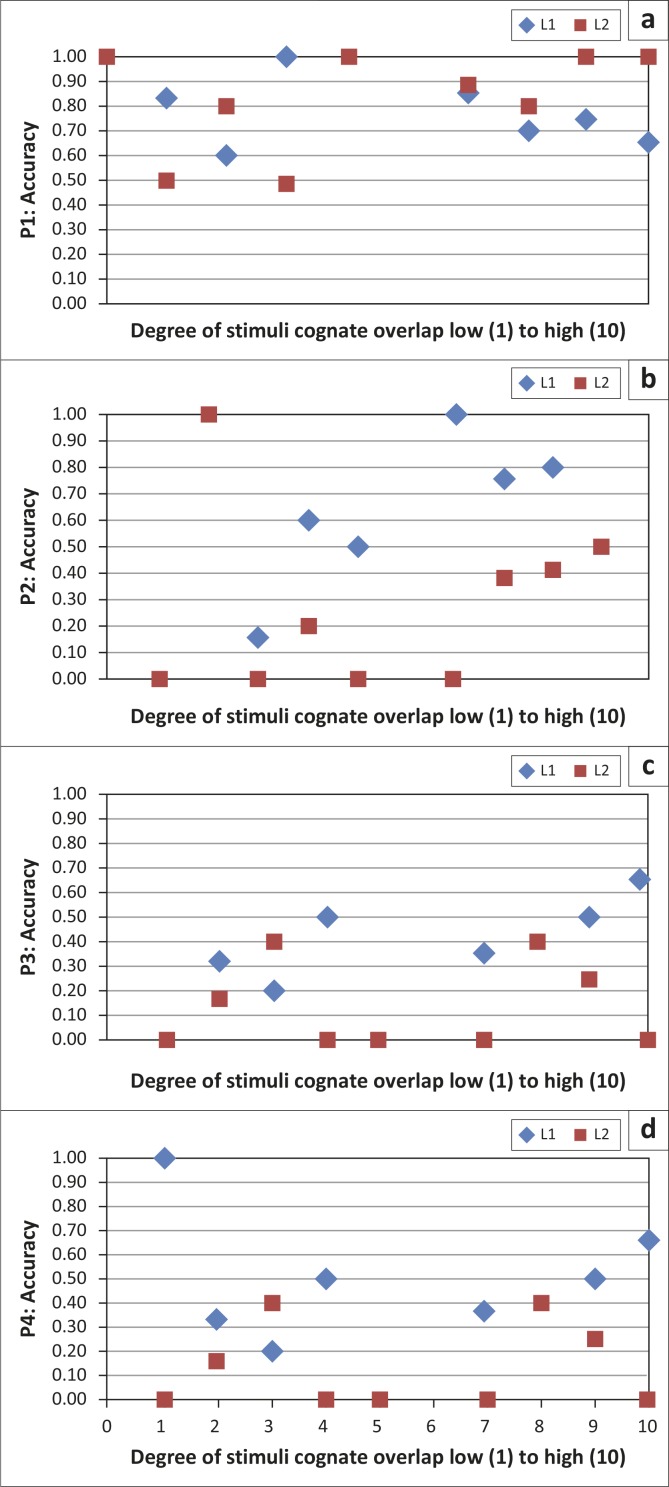
Individual scatterplots for Participants 1–4. L1 and L2 naming accuracy (y-axis) x cognate categories.

The third research question asked if any differences were observed in spoken word accuracy between L1 and L2 for high, medium and low overlapping cognates. As a reminder, out of 40 words there were 17 with high overlap, 9 with medium overlap and 14 low-overlapping cognate words. Data are displayed in Table 3 (percentage accuracy for high-overlap, medium-overlap and low-overlap cognate words) and Figure 4 (scatterplot illustrating individual performance on words according to the cognate scale 1 to 10). Whilst all L1 versus L2 comparisons for high, medium and low cognate words were non-significant, the following interesting trends were found.

**TABLE 3:** Percentage correct for L1 and L2 performance for high, medium and low overlapping cognates (Research question #3)

Participants	High overlap (*n* = 17 words)		Medium overlap (*n* = 9 words)		Low overlap (*n*= 14 words)	
	L1	L2	L1	L2	L1	L2
**P1**	0.71	0.88	0.89	0.89	0.71	0.71
**P2**	0.65	0.35	0.67	0.33	0.43	0.14
**P3**	0.47	0.33	0.33	0.00	0.29	0.21
**P4**	0.47	0.29	0.33	0.00	0.29	0.21

In regard to naming accuracy for the high overlap words, P1 showed similar performance between L1 (71%) and L2 (88%) (*p* = 0.22). P2 was higher in L1 (65%) than L2 (35%) (*p* = 0.09). P3 was slightly higher in L1 (47%) than L2 (33%) (*p* = 0.30). P4 was more accurate in L1 (47%) than in L2 (29%) (*p* = 0.30).

Naming accuracy for the medium overlap words for P1 showed equivocal performance between L1 (89%) and L2 (89%) (*p* = 1.0). P2 was higher in L1 (67%) than L2 (33%) (*p* = 0.18). P3 performed slightly higher in L1 (33%) than L2 (0%) (*p* = 0.08). P4 was slightly more accurate in L1 (33%) than in L2 (0%) (*p* = 0.08).

Naming accuracy for the low overlap words for P1 showed similar performance for L1 (71%) and L2 (71%) (*p* = 1.0). P2 was higher in L1 (43%) than L2 (14%) (*p* = 0.10). P3 performed higher in L1 (29%) than L2 (21%) (*p* = 0.68). P4 was slightly more accurate in L1 (29%) than in L2 (21%) (*p* = 0.68).

## Discussion

The purpose of this retrospective study was to investigate lexical retrieval abilities from four bilingual (Afrikaans/English) individuals with aphasia with the goal of elucidating psycholinguistic mechanisms involved in bilingual word production. Overall, this study showed that whilst all participants had a diagnosis of aphasia, their naming impairments were reflective of their reported relative use patterns prior to their stroke. These findings are consistent with the hierarchical model (Kroll & Stewart, 1994), which states that connectivity between semantic and lexical representations of the more proficient language is stronger (relative to the less proficient language), resulting in higher accuracy of naming performance post stroke. Similar patterns have also been reported in normal bilingual speakers (e.g. Edmonds & Donovan, 2014; Gollan *et al.*, 2007) and with persons with bilingual aphasia (e.g. Edmonds & Kiran, 2006).

### Accuracy

The first research question explored differences in accuracy of confrontation naming between L1 and L2. Only one participant (P1) appeared to be relatively balanced in use and proficiency in both languages and he is the only participant who did not show significant differences in accuracy across languages. On the other hand, although P2, P3 and P4 acquired both languages relatively young (≤10 years old) and demonstrated the most severe naming impairments of the group, they showed significant differences in accuracy between L1 and L2. The lower performance of these three participants, then, could be interpreted from the perspective of premorbid use patterns (and potentially proficiency levels) as opposed to severity of aphasia. Although the amount of detail we have regarding the participants’ premorbid language use is limited, our findings are consistent with Pitre’s (1895, cited in LeBrun, 1995) law, which posits that premorbid use and proficiency influence relative impairment across languages post-stroke.

The premorbid proficiency and use patterns of P2, P3 and P4 assume weaker connections between the shared L1/L2 semantic system and independent (or language-specific) L2 lexical representations (see Figure 1). Conversely, P1, who was relatively balanced, appeared to show equal connectivity between shared L1/L2 semantics and shared L1/L2 lexical representations, resulting in a lack of significance between language differences.

### Errors

The second research question explored if there were any differences in the types of errors produced in L1 versus L2. The results revealed that the proportion of semantic, phonological and mixed, omission, cross-linguistic and neologistic errors was essentially the same, regardless if the words were being spoken in L1 or in L2 (excepting P1’s semantic errors and P4’s cross-linguistic errors). From the perspective of bilingual word processing, the linguistic system is shared between languages and, as such, a linguistic impairment would evidence similarly in both languages. So, if there is a decrease in activation of the semantic system, the spread of activation is target language nonspecific, resulting in similar errors in both languages (Costa, 2005; Edmonds & Kiran, 2006). Thus, the level of linguistic impairment would not necessarily be different across languages, which appears to be the case in our participants. However, we did observe some impact of relative proficiency, which interacted with aphasia severity, on error types. P1 had more semantic substitutions in L2 relative to L1 (and also relative to the other three participants) that is likely attributed to his milder form of anomia. The presence of semantic errors indicates residual (albeit impaired) lexical or semantic activation and has been labelled ‘smart errors’ (Dell *et al.*, 1997). In Dell’s (1986) model, semantic errors are said to occur due to a competing node receiving levels of activation greater than the target node. Therefore, semantically related substitutions (e.g. apple à pear) occur during lexical access when a semantically related lexeme activation level is higher than that of the target. Omission errors, on the other hand, may occur when semantic nodes do not reach an adequate level of threshold necessary for spread of activation.

Omission errors were evidenced by P2, P3 and P4, who exhibited a more severe form of anomia. The higher proportion of omission errors (relative to other error types as well as relative to milder P1) could be attributed to the combined effect of lower proficiency or usage and an overall decrease of lexical or semantic activation. Finally, P4 showed more cross-linguistic errors in L2 versus L1 (and relative to all other participants). A cross-linguistic error is not a true omission (lack of activation in both languages); rather, it may evidence that she was relying on L1 when she was unable to produce a word in L2.

With respect to the phonological errors, P1, the only participant to show more phonological errors in L2, had the highest accuracy overall and was closer to the target in general across all attempts. Further, his phonological errors occurred in the low overlap items, suggesting that these words might be most sensitive in L2, even for relatively balanced bilinguals. P4 showed more phonologic errors in L1 (six), compared to L2 (two) which may indicate that since a phonologic error is a ‘smart’ error, it would occur in L1 and not L2 (which is the less accurate or proficient language).

### Cognate effect

The third research question explored L1 and L2 differences as a function of cognate status. This issue was important, as degree of phonological and orthographic overlap between languages has been shown to impact word processing in bilinguals (Costa *et al.*, 2000). That is, the more overlap, the faster and more accurately words are processed due to the redundant top-down and bottom-up processes. Overall, whilst our findings were statistically insignificant, the trend in the accuracy data supported the notion that regardless of severity of the participants, and for all participants except P1 who was balanced, there was a trend of higher accuracy for high cognate words compared to low overlap words. It is important to note that items were not controlled for other psycholinguistic variables. That said, there were no differences in syllable length between the high and low cognate word lists.

P1 showed a slightly higher score for cognates in L2 (88% accuracy) compared to L1 (71%). Although not statistically significant, this trend is consistent with Roberts and Deslauriers (1999), who showed higher accuracy for cognates in the L2 of highly balanced English/French bilingual speakers with aphasia, a pattern also observed in normal bilingual speakers (Gollan *et al.*, 2007; Roberts & Deslauriers, 1999). Interestingly, P1 showed equal accuracy in medium and low overlap words, indicating that high overlap words (compared to medium and low overlap) facilitate processing due to the shared semantic and phonological activation.

The participants with a higher performance in one language did not show the same effect of cognates as P1, though an effect was seen. In these participants, picture naming in their L1 was slightly higher than L2 in high, medium and low phonological overlap words, suggesting that cognates helped retrieval in both languages, but with a consistent trend of higher naming in L1. This is consistent with the multilingual participant described by Stadie *et al.* (1995).

Also, the higher performance seen by the three participants in their more proficient language indicates that words with overlapping cognates share semantic and phonological activation from two sources (target and nontarget language representations), thereby facilitating production. The converse is true as well. A trend of lower accuracy observed with the low overlap words spoken with the least proficient language supports this notion as well.

Finally, phonological overlap may indeed be a good way to characterise words for bilinguals in addition to more conventional, psycholinguistic terms, such as frequency (Edmonds & Donovan, 2012, 2014). From a clinical standpoint, training items with high cognate status may indeed be an efficient approach, since this would activate direct connections between semantics and phonology within languages and bidirectionally activate phonology as well. Such a manipulation may result in more cross-linguistic generalisation for the trained words regardless of whether the more or less proficient language is trained (Kohnert, 2004) (as compared to training words without phonological overlap, e.g. Edmonds & Kiran, 2006; but, see Kurland & Falcon, 2011), which would be particularly beneficial when a clinician does not share the second language with the patient. However, it should be noted that generalisation to untrained (noncognate) words may still require direct training in both languages (e.g. Edmonds & Kiran, 2006; Kohnert, 2004) and many languages do not have a high incidence of cognates (Kohnert, 2009).

### Limitations and future directions

There are several limitations that are important to note. Firstly, the sample size was small. There were only four participants and additional persons who are Afrikaans/English speaking with aphasia need to be tested to replicate these findings. Secondly, there is limited language use information known on these individuals other than their brief report. Thirdly, the stimulus items employed in this study were not selected for the cognate analysis. Fourthly, because there are no available standardised aphasia testing measures for English/Afrikaans, overall language severity of this particular sample is unknown.

In the future, items should be selected based on specific psycholinguistic variables of interest. Also, since the connections between the languages of bilingual persons are fluid and may indeed change depending on use, issues pertaining to the impact of the language in which therapy is provided as well as the impact of language usage post-stroke (e.g. individuals unable to return to work who are thus no longer exposed as much to one of the languages) on patterns of recovery should be addressed in future research studies. Finally, it is important to note that parallels to other African languages are limited as the results will be person-specific and may depend on the classification of the languages that are compared. Both languages in the current study were Germanic languages. The clinical implications for this study are that the clinician needs to gather necessary information during the assessment period on level of proficiency and use. Also, if possible, the clinician should evaluate the word retrieval impairment across languages to provide additional information regarding severity.

## Conclusions

Characterising phonological word equivalents across languages in word evaluations (i.e. translations) in evaluations of lexical retrieval in aphasia may provide additional insight into the interactions between premorbid proficiency and use, level of impairment and degree of phonological overlap across languages.

## References

[CIT0001] Barber C. (1999). *The English language: A historical introduction.* Cambridge, UK: Cambridge University Press.

[CIT0002] Costa A. (2005). Lexical access in bilingual production. In Kroll J.F. & de Groot A.M.B. (Eds.), *Handbook of bilingualism: Psycholinguistic approach* (pp. 308–325). New York, NY: Oxford University Press.

[CIT0003] Costa A., Caramazza A., & Sebastian-Galles N. (2000). The cognate facilitation effect: Implications for models of lexical access. *Journal of Experimental Psychology: Learning, Memory, and Cognition*, 26, 1283–1296. http://dx.doi.org/10.1037/0278-7393.26.5.1283 10.1037//0278-7393.26.5.1283 11009258

[CIT0004] Costa A., Santesteban M., & Caño A. (2005). On the facilitatory effects of cognate words in bilingual speech production. *Brain and Language*, 94, 94–103. http://dx.doi.org/10.1016/j.bandl.2004.12.002 15896387 10.1016/j.bandl.2004.12.002

[CIT0005] De Groot A.M.B. (1992). Determinants of word translation. *Journal of Experimental Psychology: Learning, Memory, and Cognition*, 18, 1001–1018. http://dx.doi.org/10.1037/0278-7393.18.5.1001

[CIT0006] Dell G.S. (1986). A spreading activation theory of retrieval in sentence production. *Psychological Review*, 93, 283–321. http://dx.doi.org/10.1037/0033-295X.93.3.283 3749399

[CIT0007] Dell G.S., Schwartz M.F., Martin N.M., Saffran E.M., & Gagnon D.A. (1997). Lexical access in aphasic and nonaphasic speakers. *Psychological Review*, 104, 801–838. http://dx.doi.org/10.1037/0033-295X.104.4.801 9337631 10.1037/0033-295x.104.4.801

[CIT0008] Edmonds L.A., & Donovan N.J. (2012). Item-level psychometrics and predictors of performance for Spanish/English bilingual speakers: An object and action naming battery. *Journal of Speech, Language, and Hearing Research*, 55, 359–381. http://dx.doi.org/10.1044/1092-4388(2011/10-0307) 10.1044/1092-4388(2011/10-0307) 22215032

[CIT0009] Edmonds L.A., & Donovan N.J. (2014). Research applications for an object and action naming battery to assess naming skills in adult Spanish-English bilingual speakers. *Behavior Research Methods*, 46, 456–471. http://dx.doi.org/10.3758/s13428-013-0381-7 24002987 10.3758/s13428-013-0381-7

[CIT0010] Edmonds L., & Kiran S. (2006). The effect of semantic naming treatment on crosslinguistic generalization in bilingual aphasia. *Journal of Speech, Language, and Hearing Research*, 49, 729–748. http://dx.doi.org/10.1044/1092-4388(2006/053) 10.1044/1092-4388(2006/053) 16908872

[CIT0011] Ferrand L., & Humphreys G.W. (1996). Transfer of refractory states across languages in a global aphasic patient. *Cognitive Neuropsychology*, 13(8), 1163–1191. http://dx.doi.org/10.1080/026432996381692

[CIT0012] Gollan T.H., & Acenas L.R. (2004). What is a TOT? Cognate and translation effects on tip-of-the-tongue states in Spanish-English and Tagalog-English bilinguals. *Journal of Experimental Psychology: Learning, Memory and Cognition*, 30, 246–269. http://dx.doi.org/10.1037/0278-7393.30.1.246 10.1037/0278-7393.30.1.246 14736310

[CIT0013] Gollan T.H., Fennema-Notestine C., Montoya R.I., & Jernigan T.L. (2007). The bilingual effect on Boston Naming Test performance. *Journal of the International Neuropsychological Society*, 13, 197–208. http://dx.doi.org/10.1017/s1355617707070038 17286875 10.1017/S1355617707070038

[CIT0014] Grosjean F. (1998). Studying bilinguals: Methodological and conceptual issues. *Bilingualism: Language and Cognition*, 1(2), 131–149. http://dx.doi.org/10.1017/S136672899800025X

[CIT0015] Kohnert K. (2004). Cognitive and cognate-based treatments for bilingual aphasia: A case study. *Brain and Language*, 91, 294–302. http://dx.doi.org/10.1016/j.bandl.2004.04.001 15533555 10.1016/j.bandl.2004.04.001

[CIT0016] Kohnert K. (2009). Cross-language generalization following treatment in bilingual speakers with aphasia: A review. *Seminars in Speech and Language*, 30, 174–186. http://dx.doi.org/10.1055/s-0029-1225954 19711235 10.1055/s-0029-1225954

[CIT0017] Kohnert K., Windsor J., & Miller R. (2004). Crossing borders: Recognition of Spanish words by English-speaking children with and without language impairment. *Applied Psycholinguistics*, 25, 543–564. http://dx.doi.org/10.1017/S0142716404001262

[CIT0018] Kroll J.F., & Stewart E. (1994). Category interference in translation and picture naming: Evidence for asymmetric connections between bilingual memory representations. *Journal of Memory and Language*, 33, 149–174. http://dx.doi.org/10.1006/jmla.1994.1008

[CIT0019] Kuiper K., & Scott Allan W. (1996). *An introduction to English language: Sound, word and sentence.* London, UK: MacMillan Press.

[CIT0020] Kurland J., & Falcon M. (2011). Effects of cognate status and language of therapy during intensive semantic naming treatment in a case of severe nonfluent bilingual aphasia. *Clinical Linguistics & Phonetics*, 25, 584–600. http://dx.doi.org/10.3109/02699206.2011.565398 21631308 10.3109/02699206.2011.565398

[CIT0021] LeBrun Y. (1995). The study of aphasia in bilingual or multilingual speakers: Pitres’ legacy. In Paradis M. (Ed.), *Aspects of aphasia in bilingual or multilingual speakers* (pp. 11–21). Oxford, UK: Elsevier Science Ltd.

[CIT0022] Odendaal F.F. (1989). Afrikaanse fonetiek. In Botha T.J.R. (Ed.), *Inleiding tot die Afrikaanse taalkunde [Introduction to Afrikaans linguistics]* (2nd edn., pp. 155–188). Pretoria, South Africa: Academica.

[CIT0023] Roberts P.M., & Deslauriers L. (1999). Picture naming of cognates and non-cognate nouns in bilingual aphasia. *Journal of Communication Disorders*, 32, 1–23. http://dx.doi.org/10.1016/S0021-9924(98)00026-4 9921457 10.1016/s0021-9924(98)00026-4

[CIT0024] Stadie N., Springer L., De Bleser R., & Burk F. (1995). Oral and written naming in a multilingual patient. In Paradis M. (Ed.), *Aspects of bilingual aphasia* (pp. 85–100). New York, NY: Elsevier Science.

[CIT0025] Statistics South Africa. (2012). *Census 2011: Census in brief.* Retrieved May 19, 2015, from http://www.statssa.gov.za/census/census_2011/census_products/Census_2011_Census_in_brief.pdf

[CIT0026] Thompson L.M. (2001). *A history of South Africa.* (3rd edn.). New Haven, CT: Yale University Press http://dx.doi.org/10.1080/02582470108671409

